# The Renal Effects of Vanadate Exposure: Potential Biomarkers and Oxidative Stress as a Mechanism of Functional Renal Disorders—Preliminary Studies

**DOI:** 10.1155/2014/740105

**Published:** 2014-01-28

**Authors:** Agnieszka Ścibior, Dorota Gołębiowska, Agnieszka Adamczyk, Irmina Niedźwiecka, Emilia Fornal

**Affiliations:** ^1^Laboratory of Physiology and Animal Biochemistry, Department of Zoology and Invertebrate Ecology, Institute of Environmental Protection, The John Paul II Catholic University of Lublin, 102 Kraśnicka Avenue, 20-718 Lublin, Poland; ^2^Laboratory of Oxidative Stress, Center for Interdisciplinary Research, The John Paul II Catholic University of Lublin, 102 Kraśnicka Avenue, 20-718 Lublin, Poland; ^3^Laboratory of Separation and Spectroscopic Method Applications, Center for Interdisciplinary Research, The John Paul II Catholic University of Lublin, 102 Kraśnicka Avenue, 20-718 Lublin, Poland

## Abstract

The alterations in the levels/activities of selected biomarkers for detecting kidney toxicity and in the levels of some oxidative stress (OS) markers and elements were studied in male rats to evaluate biochemically the degree of kidney damage, investigate the role of OS in the mechanism of functional renal disorders, reveal potential biomarkers of renal function, and assess the renal mineral changes in the conditions of a 12-week sodium metavanadate (SMV, 0.125 mg V/mL) exposure. The results showed that OS is involved in the mechanism underlying the development of SMV-induced functional renal disturbances. They also suggest that the urinary cystatin C (CysC_u_) and kidney injury molecule-1 (KIM-1_u_) could be the most appropriate to evaluate renal function at the conditions of SMV intoxication when the fluid intake, excreted urinary volume (EUV), body weight (BW), and the urinary creatinine excretion (Cre_u_) decreased. The use of such tests as the urinary lactate dehydrogenase, alkaline phosphatase, **γ**-glutamyltranspeptidase, and N-acetyl-**β**-D-glucosaminidase (LDH_u_, ALP_u_, GGTP_u_, and NAG_u_) seems not to be valid given their reduced activities. The use of only traditional biomarkers of renal function in these conditions may, in turn, be insufficient because their alterations are greatly influenced by the changes in the fluid intake and/or BW.

## 1. Introduction

Vanadium (V) is a well-known powerful prooxidant. It may modify oxidative stress (OS) in the cells and be involved in oxidative injury mechanisms [1]. Its prooxidant action has been demonstrated in *in vivo* and *in vitro* conditions [2–7]. It has been shown that the free radical process-lipid peroxidation (LPO), which is a biochemical biomarker of cellular dysfunction and an index of cytotoxicity [8], is enhanced by V in the kidney [3, 8]. It has also been suggested that LPO may be predictive of renal dysfunction [8].

Kidney is particularly vulnerable to deleterious effects of V. The susceptibility of this organ to V may be a reflection of its accumulation in this tissue [8]. It has been reported that V may be present in tubular cells in a readily exchangeable form as well as in low and high molecular mass complexes, and it may be excreted free or bound to proteins after prolonged exposure [9, 10]. It has also been speculated that V may be involved in the pathogenesis of distal renal tubular acidosis (dRTA), renal stone disease, “uremic syndrome,” and acquired cystic kidney disease [11–13]. A suggestion that prolonged intake of high-dose V supplements may cause serious kidney toxicity has been put forward as well [14, 15].

The occupational and environmental toxicological impact of V and the fact that kidneys are critical for its poisoning [16–19], the modest number of biomarkers of possible functional renal disturbances under vanadate exposure examined until now, and the insufficient information about the contribution of OS in the mechanism underlying the vanadate-induced functional kidney disorders prompted us to answer the following questions. (a) To what extent do the effects of 12-week sodium metavanadate (NaVO_3_, SMV, 0.125 mg V/mL) exposure alter the levels/activities of some biomarkers of renal toxicity in rats? We intended to examine traditional biomarkers classified as not very specific or sensitive but routinely used in the diagnosis of kidney function and those that allow distinguishing, to some degree, structural and functional renal disorders and have a potential for determining the site of renal tubular damage [20–22]. (b) To what degree do the effects of SMV exposure change the homeostasis of some micro- and macroelements in the kidney? (c) Which of the biomarkers examined are the most sensitive in our experimental conditions? (d) What value/information do some biomarkers add to the existing data? (e) Is OS involved in the mechanism of the development of kidney function disorders during SMV intoxication? (f) Are there any significant relationships between the measured variables? Since the measurement of the activities of some enzymes in tissues and biological fluids may play a significant role in detection of tissue cellular injury and point to damage long before histological alterations, both cytosolic and lysosomal enzymes as well as those located on the brush-border membrane have been taken into consideration and illustrated in the present report.

## 2. Materials and Methods

### 2.1. Reagents

The kits for determination of the plasma concentrations of TP_p_, U_p_, UA_p_, Mg_p_, and Ca_p_ and the urinary levels of U_u_, UA_u_, and Cre_u_ were obtained from Emapol (Gdańsk, Poland), whereas the kits for determination of the plasma Cu_p_ and Zn_p_ levels were purchased from Sentinel Ch. kits (Milan, Italy). The reagents for determination of the levels/activities of the plasma (Cre_p_, ALB_p_, LDH_p_, GGTP_p_, and ALP_p_), urinary (ALB_u_, TP_u_, LDH_u_, GGTP_u_, and ALP_u_), and renal (LDH_k_, GGTP_k_, and ALP_k_) biomarkers as well as the reagent for measurement of the levels of electrolytes in the plasma and urine (Na_p/u_, K_p/u_, and Cl_p/u_) were acquired from Alpha Diagnostics (Warsaw, Poland). The kit for estimation of total antioxidant status in the kidney (TAS_k_) was bought from Calbiochem-Novabiochem Corporation (San Diego, CA, USA), whereas the kits for determination of the urinary levels of NAG_u_ (E90069Ra), CysC_u_ (E90896Ra), *β*_2_M_u_ (E90260Ra), and KIM-1_u_ (E90785Ra) and of the plasma concentrations of CysC_p_ (E90896Ra) and (*β*_2_M_p_ E90260Ra) were derived from Uscn Life Science Inc. (Wuhan, China). NaVO_3_, Triton X-100, and the caesium chloride lanthanum chloride buffer (CsClLaCl) were purchased from Sigma Chemical (St. Louis, USA). Nitric acid (HNO_3_, 65% suprapure) and diethyl ether (C_4_H_10_O) were acquired from Merck (Darmstadt, Germany), whereas hydrogen peroxide (H_2_O_2_; 30% pure P.A.) and the physiological buffered saline (PBS) were purchased from POCH (Gliwice, Poland) and from the Serum and Vaccine Factory (Biomed, Lublin, Poland), respectively. Stocks of V, Mg, Zn, and Cu (Inorganic Ventures, Christiansburg, USA) and a stock of Ca (Spectracer, UK) atomic absorption standard solutions as well as a multielement standard solution for Na and K (Sigma-Aldrich) were used in the element analysis by the Atomic Absorption Spectrometry (AAS) method. Ultrapure water was received from an ultrapure water HLP Spring 5R system ^1^ (Hydrolab, Gdańsk, Poland). All the chemicals were of the highest quality available.

### 2.2. Animals and Experimental Design

The experiment was conducted according to the experimental protocol approved by the 1st Local Ethical Committee for Animal Studies in Lublin. Biological material used in this study originated from some of the outbred albino male Wistar rats used in the previous study [23]. The animals were kept in an animal room with controlled conventional conditions (one rat per stainless steel cage) and received, inter alia, deionised water (Group I, Control, 8 rats) or a water solution of NaVO_3_ at a concentration of 0.125 mg V/mL (Group II, SMV, 8 rats) to drink in special bottles with the scale every day over a 12-week period. All the rats had *ad libitum* access to fresh deionised water, SMV solution, and a standard rodent diet (Labofeed B, Fodder and Concentrate Factory, Kcynia, Poland) in which the concentration of V had been assessed by Graphite Furnace AAS (GF-AAS) in our laboratory, and it was about 0.17 *μ*g V/g. More details about the chow had been provided by us previously [23]. The intake of food, water, and SMV solution was monitored daily and body weight (BW) was obtained weekly. The water and SMV solution intake was expressed as mL/rat/24 h, whereas the food intake was expressed as g/rat/24 h. The daily V intake in the SMV-exposed rats was estimated based on the 24 h consumption of the drinking SMV solution and expressed as mg V/kg b. wt./24 h. During the whole experiment, the animals were observed in order to assess their general health. The doses of V presented in the study are within the broad dose range that was used for demonstrating the antidiabetic activity of V [19, 24] and for analysing its pharmacokinetic behaviour [25] on an animal model. The concentration of V in rats' urine determined in the present study may reflect exposure to this element occurring especially in persons occupationally exposed to this metal [26, 27].

Every second week in the course of the experiment and in the 11th week, 24-hour urine was collected from each rat placed individually in plastic metabolic cages not equipped with a cooling system (Tecniplast, Italy), which allowed separate collection of urine and faeces. During that time, each rat had access to food and water or the SMV solution. The urine samples from all the control and SMV-exposed rats were used immediately for determination of some biochemical parameters and for measurements of excreted urine volume (EUV) and urine pH. The volumes of excreted 24-hour urine were measured with a measuring cylinder, whereas urine pH and blood in the urine were tested on the H-100 urine analyser (DIRUI, China) using urinary dipsticks. Portions of the urine samples that were not used immediately were frozen at −80°C in a deep-freezer HFU 486 Basic^1^ (Thermo Fisher Scientific, Germany) and stored until further analyses.

All the rats were sectioned at the end of week 12. Whole blood was taken from the jugular vein into plastic tubes with heparin as an anticoagulant under anaesthesia with ketamine/xylazine cocktail (100 mg/mL and 20 mg/mL, resp., i.p.) and centrifuged (5 min, 1500 ×g, 4°C). Plasma portions were collected for routine analyses of clinical chemistry parameters and for other biological determinations. The kidneys and femurs (right and left) were immediately removed. The kidneys were washed in ice-cold physiological saline (0.9% NaCl) and weighted. The right femurs (after removal of the overlying tissue with stainless steel knives) were also washed in 0.9% NaCl, weighed, and stored frozen at –80°C until the time of bone digestion. Before digestion, all the collected right femurs were cut using a diamond-disk saw (Metkon Micracut 175)^1^, cooled with ultrapure water to separate the proximal and distal femoral epiphysis (PFE and DFE) (a region of trabecular bone) from the femoral diaphysis (FD) (a region of cortical bone). Next, bone marrow was removed from all the FDs, which then were first soaked with ether (to remove fat content) and later in H_2_O_2_ (to remove remaining blood deposits). Afterwards, the cleaned FDs were washed in ice-cold PBS and then in ultrapure water. Next, they were dried at room temperature into constant mass. The cleaned and bone marrow-deprived FD samples (~0.355 g, mean) were used for digestion.

### 2.3. Decomposition of the Kidney, Urine, and FD

In order to determine Mg, Ca, V, Zn, Cu, Na, and K in the kidney and Mg, Ca, V, Zn, and Cu in the urine, 0.5 g of kidney and 1 mL of urine were wet-mineralized with 5 mL of 65% HNO_3_ in 12 Teflon Fluor Modified (TFM) closed digestion vessels using a model Speedwave Four microwave digestion system^1^ (Berghof, Germany) equipped with a temperature and pressure sensor in each vessel. In turn, ~0.355 g of FD was wet-mineralized in the same digestion system but in the presence of 5 mL of 65% HNO_3_ and 1 mL of 30% H_2_O_2_ to determine V, Mg, and Ca. Before the measurements, all the decomposed samples of kidney, FD, and urine were transferred into 25 mL volumetric polypropylene flasks by washing the inner surface of the digestion vessels with ultrapure water three times and filled up to the mark with ultrapure water. Next, determinations of the selected elements were performed by the AAS method.

### 2.4. Determination of Some Renal Biomarkers

**Figure 1:**
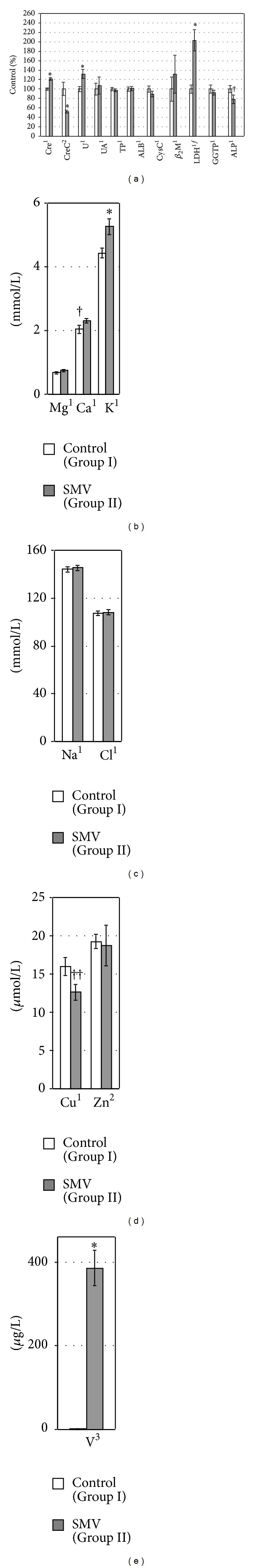
The levels/activities of some biomarkers of renal toxicity (a) and the concentrations of selected elements ((b)–(e)) in rat plasma. ^1,2,3^Data were tested by Student's *t*-test, Welch's *t*-test, or Mann-Whitney's *U* test, respectively. *Significant differences, compared with the Control (Group I). ^*f*^Logarithmically transformed data. ^††, †^ *P* = 0.05, *P* = 0.08, respectively, compared with the Control (Group I).

**Figure 2:**
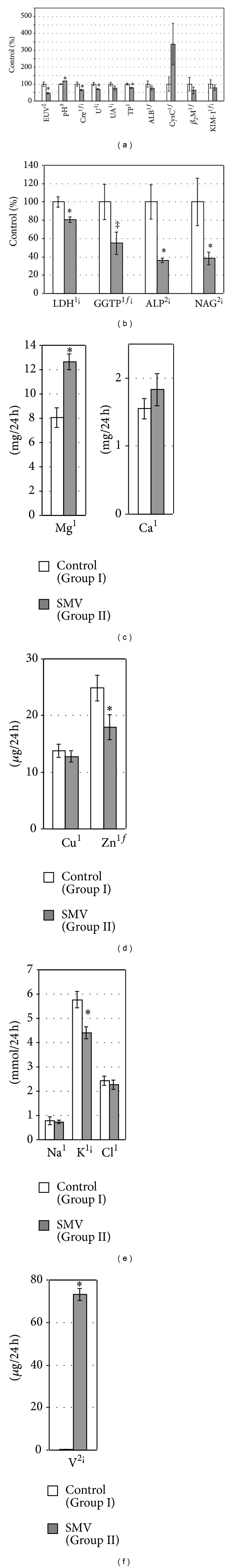
EUV, urine pH, and the urinary levels/activities of some biomarkers of renal toxicity (a and b) and the urinary levels of selected elements ((c)–(f)) normalized per 24-hour diuresis in the tested rats. ^1,2,3^Data were tested by Student's *t*-test, Welch's *t*-test, or Mann-Whitney's *U* test, respectively. *Significant differences, compared with the Control (Group I). ^*f*^Logarithmically transformed data. ^¡^Correlated with excreted urinary volume (EUV). ^‡^ *P* = 0.06, compared with the Control (Group I).

**Figure 3:**
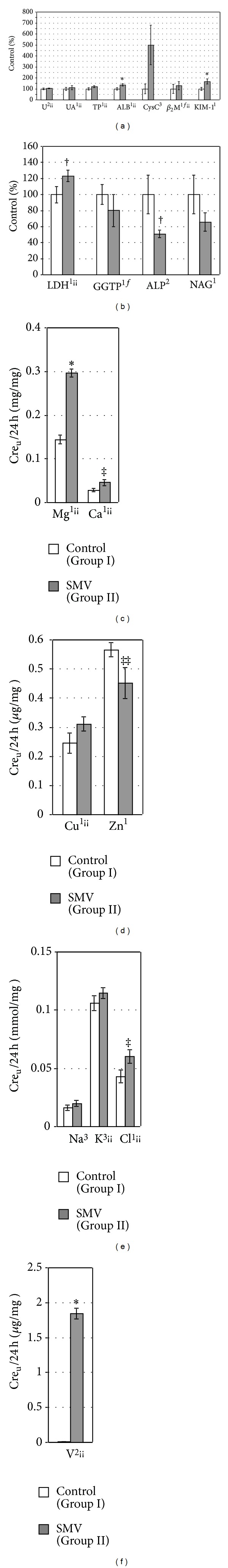
The urinary levels/activities of some biomarkers of renal toxicity ((a) and (b)) and the urinary levels of selected elements ((c)–(f)) normalized per 24-hour urinary Cre_u_ excretion in the tested rats. ^1,2,3^Data were tested by Student's *t*-test, Welch's *t*-test, or Mann-Whitney's *U* test, respectively. *Significant differences, compared with the Control (Group I). ^*f*^Logarithmically transformed data. ^¡¡^Correlated with the urinary Cre_u_ excretion. ^‡, ‡‡, †^ *P* = 0.06, *P* = 0.07, and *P* = 0.08, respectively, compared with the Control (Group I).

**Figure 4:**
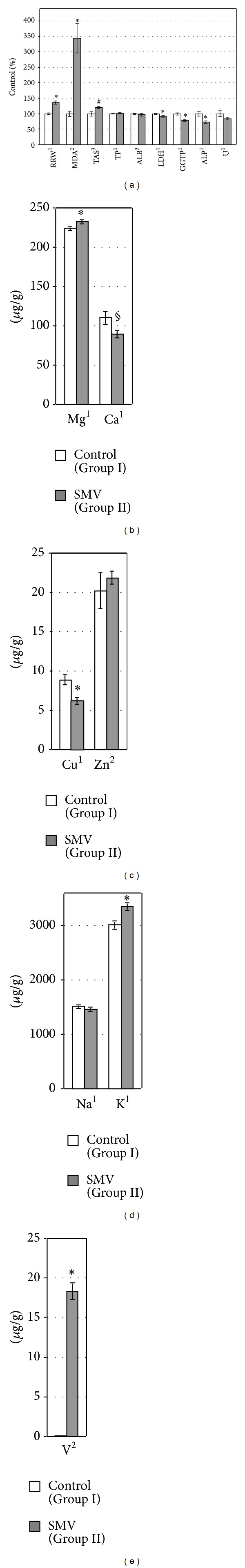
Renal relative weight (RRW), the levels/activities of some OS markers, proteins, and enzymes (a) in the kidney, and the concentrations of selected elements ((b)–(e)) in the same organ of the tested rats. ^1,2,3^Data were tested by Student's *t*-test, Welch's *t*-test, or Mann-Whitney's *U* test, respectively. *Significant differences, compared with the Control (Group I). ^#, §^ *P* = 0.09, *P* = 0.05, respectively, compared with the Control (Group I).

The concentrations of U_p_, UA_p_ and TP_p_ in the plasma as well as U_u_, UA_u_, and Cre_u_ in 24-hour urine were determined colourimetrically by measuring the absorbance with a Thermo Spectronic BioMate5 UV-VIS spectrophotometer (UK). The concentrations of ALB_p_, ALB_u_, TP_u_, and Cre_p_ and the activities of LDH_p_, GGTP_p_, ALP_p_, LDH_u_, GGTP_u_, and ALP_u_ in the plasma and urine as well as the activities of LDH_k_, GGTP_k_, and ALP_k_ in the kidney were measured using an automatic biochemical analyser BS-120^1^ (Mindray, China). The levels of CysC_p_ and *β*_2_M_p_ in the plasma and the levels of CysC_u_, *β*_2_M_u_, KIM-1_u_, and NAG_u_ in the urine were assessed by a traditional method using rat-specific commercial enzyme-linked immunosorbent assay (ELISA) kits and an ELISA microplate reader Synergy 2^1^ equipped with an automated microplate strip washer ELx50^1^ and a microplate shaker^1^ (BIO-TEK Instruments Inc., USA). All the ELISA tests were performed according to the manufacturer's protocols. Before the measurements, thawed plasma and urine samples were mixed by inversion, centrifuged with cooling (1500 rpm for 10 min, 4°C, or 2000 rpm for 5 min, 4°C, resp.) using a centrifuge Heraeus Megafuge 11R^1^ (Thermo Fisher Scientific, Germany), and immediately used for analysis. The optimal factors of dilution for some samples were chosen when necessary and the samples were diluted with ultrapure water or with PBS (pH 7.4 ± 0.2). In order to evaluate the glomerular filtration rate (GFR), calculation of creatinine clearance (CreC) was performed. The results for all the above-mentioned parameters are expressed as % of the control and presented in Figures 1(a), 2(a), 2(b), 3(a), 3(b), and 4(a).

### 2.5. Determination of Elements in Biological Fluids and Tissues

#### 2.5.1. Spectrophotometric Determination of Ca, Mg, Zn, and Cu in the Plasma

The measurements of these elements were performed using a BioMate5 spectrophotometer with the direct colourimetric method according to the protocols of the kits, and their concentrations are presented in Figures 1(b) and 1(d).

#### 2.5.2. Determination of Na, K, and Cl in the Plasma and Urine

The electrolytes mentioned were measured using an automatic EasyLyte analyser Na/K/Cl^1^ (Medica). Their urinary excretion was first normalized against 24-hour diuresis and against 24-hour urinary Cre_u_ excretion, and their concentrations are presented in Figures 1(b), 1(c), 2(e), and 3(e).

#### 2.5.3. Atomic Absorption Measurements of Mg, Ca, V, Zn, Cu, Na, and K in Digested Kidney Samples, Mg, Ca, V, Zn, and Cu in Digested Urine Samples, V, Mg, and Ca in Digested FD Samples, and V in Nondigested Plasma Samples

The elements were determined by Flame or Graphite Furnace AAS (F-AAS or GF-AAS, resp.) using a SpectrAA Z-2000 TANDEM atomic absorption spectrometer^1^ (Hitachi, Japan) equipped with a Zeeman background corrector. A specific matrix modifier was used for V determination. In order to determine the V concentration in the plasma, the samples were diluted with 0.05% Triton X-100 and in ultrapure water when necessary. All the operating parameters of the instrument and the details of measurements of all the above-mentioned elements together with the values of the detection, quantification limits (LOD and LOQ, resp.), and the coefficient of variations (CV) are shown in Table 1.

**Figure 5:**
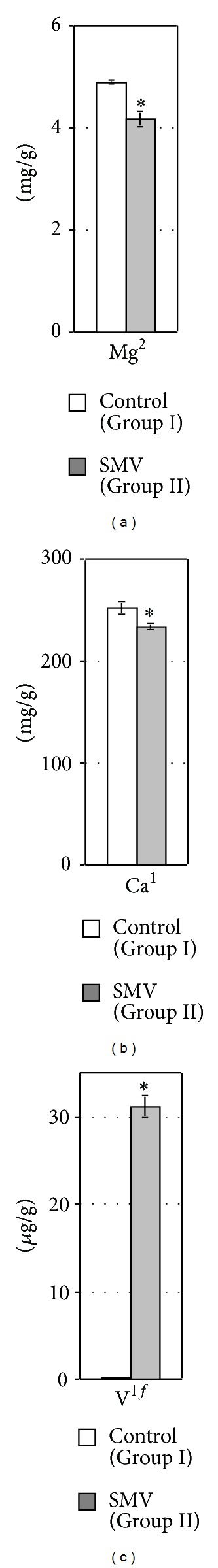
The concentration of Mg (a), Ca (b), and V (c) in the rat femoral diaphysis (FD). ^1,2^Data were tested by Student's *t*-test or Welch's *t*-test, respectively. *Significant differences, compared with the Control (Group I).

The method of standard addition was performed in order to estimate the effect of interferences during Mg assessment. Mg, Ca, V, Zn, Cu, Na, and K were determined by application of a calibration curve using working standard solutions, which were obtained from a stock atomic absorption standard solutions containing 1000 *μ*g Mg, Ca, V, Zn, Cu, Na, and K/mL by dilution with 5% (v/v) HNO_3_. The 10% CsClLaCl buffer was used to determine Ca. The analytical quality of the measurements was checked with the use of Certified Reference Materials (CRMs) such as Bovine Liver 1577 c (NIST), Seronorm Trace Elements Urine 201205 (SERO), Trace Elements in Natural Water 1640 a (NIST), and Bone Ash 1400 (NIST). The analysis of these CRMs confirmed the reliability of the proposed approach. The certified and determined values of all the elements examined in the above-mentioned CRMs are presented in Table 1. The plasma V level is presented in Figure 1(e). In turn, the urinary excretion of the above-mentioned elements investigated was also first normalized against 24-hour diuresis and against 24-hour urinary Cre_u_ excretion. This is illustrated in Figures 2(c), 2(d), 2(f), 3(c), 3(d), and 3(f). However, the renal V, Mg, Ca, Zn, Cu, Na, and K concentrations as well as the FD V, Mg, and Ca concentrations are presented in Figures 4(b)–4(e) and in Figures 5(a)–5(c).

### 2.6. Determination of Renal Lipid Peroxidation (LPO) and Total Antioxidant Status (TAS)

All details concerning the methodology of determination of LPO and the preparation of kidney homogenates and supernatants for the renal MDA_k_ and TAS_k_ measurements had already been provided [2, 3]. The results of both the OS markers mentioned are expressed as % of control and illustrated in Figure 4(a).

### 2.7. Statistical Analysis

The statistical analysis of the data was performed with the Statistica and SPSS, versions 9.0 and 14.0 PL for Windows, respectively. The normal distribution of the data was tested by Shapiro-Wilk's normality test. Grubbs' test was performed to detect the presence of outliers from a normal distribution. The homogeneity of variances was verified employing Levene's test and sometimes additionally Hartley's Fmax, Cochran's C, and Bartlett's tests. Student's *t*-test and Welch's *t*-test were applied to compare the means of two independent groups when the data met the assumptions of ANOVA and when they had a normal distribution but the variances were not homogenous, respectively. In some cases, logarithmic transformation of the data was used to *make them more normal*. In turn, the nonparametric Mann-Whitney *U* test was applied when the data did not meet the assumptions of ANOVA. The results were presented as mean ± the standard error of the mean (SEM). A *P* value less than 0.05 was taken as a criterion for a statistically significant difference. Pearson's correlation analyses were applied to assess the relationships between the measured variables. Correlations were considered statistically significant at *P* < 0.05.

## 3. Results

### 3.1. General Observation and Changes in Some Basic Parameters

In the animals exposed to SMV, no distinct differences in physical appearance and motor behaviour were observed during the 12-week experimental period, compared with the control. Some of the SMV-intoxicated rats had gastrointestinal disturbances probably caused by the consumption of the V dose. One-day diarrhoea was observed in four animals from this group in the first, second, third, or fourth week of the experiment. Only one rat from this group had three-day diarrhoea in the first week of the experiment. The fluid and food intake as well as BW (Table 2) and EUV (Figure 2(a)) in the SMV-exposed rats were lower, compared with the control animals. The dose of V consumed by the rats during the 12-week period reached the value of about 13 mg V/kg b. wt./24 h (Table 2).

### 3.2. Plasma, Urinary, and Renal Levels of the Examined Biomarkers and the Dipstick Urinalysis (Blood and pH)

The concentrations of Cre_p_ and U_p_ in the SMV-exposed rats increased (Figure 1(a)), whereas CreC (Figure 1(a)) and the levels of urinary excretion of Cre_u_ (per 24 h) and U_u_ (per 24 h) decreased significantly (Figure 2(a)), in comparison with the control animals. In turn, the urinary level of U_u_ (per Cre_u_/24 h) (Figure 3(a)) and the concentration of U_k_ in the kidney (Figure 4(a)) were unaltered. The levels of UA_p_ (Figure 1(a)) and UA_u_ (per 24 h and per Cre_u_/24 h, Figures 2(a) and 3(a), resp.) also remained unchanged in response to the SMV exposure.

The levels of TP_p_ and ALB_p_ in the SMV-exposed rats did not alter significantly, compared with the control (Figure 1(a)), but the urinary excretion of TP_u_ (per 24 h) and ALB_u_ (per 24 h) decreased and unchanged, respectively, (Figure 2(a)). On the contrary, the urinary levels of TP_u_ (per Cre_u_/24 h) in the same group of rats were unaltered and ALB_u_ (per Cre_u_/24 h) increased, in comparison with the control (Figure 3(a)). In turn, the renal concentration of TP_k_ and ALB_k_ did not change markedly (Figure 4(a)).

The concentrations of CysC_p_ and *β*_2_M_p_ remained unaltered between the groups (Figure 1(a)). However, the urinary excretion of CysC_u_ (per 24 h, Figure 2(a), and per Cre_u_/24 h, Figure 3(a)) increased markedly but the urinary *β*_2_M_u_ level (per 24 h, Figure 2(a), and per Cre_u_/24 h, Figure 3(a)) was unchanged. In turn, the urinary level of KIM-1_u_ (per 24 h, Figure 2(a), and per Cre_u_/24 h, Figure 3(a)) in the SMV-intoxicated rats did not alter and increased, respectively, compared with the control.

The activity of LDH_p_ increased, ALP_p_ decreased, and GGTP_p_ was unchanged in the rats after the SMV intoxication, in comparison with the control (Figure 1(a)). In turn, the urinary activities of LDH_u_, GGTP_u_, and ALP_u_ (per 24 h) as well as the urinary activities of GGTP_u_ and ALP_u_ (per Cre_u_/24 h) and NAG_u_ (per 24 h and per Cre_u_/24 h) were lowered in the SMV-exposed rats, compared with the control (Figures 2(b) and 3(b)). However, the urinary activity of LDH_u_ (per Cre_u_/24 h) only showed a visible trend toward an increase (Figure 3(b)). In turn, the renal activity of LDH_k_, GGTP_k_, and ALP_k_ in the SMV-intoxicated rats was lowered, in comparison with the control (Figure 4(a)).

Urine dipstick analysis did not reveal presence of blood in the urine of the SMV-intoxicated rats (data not shown). However, the urine pH in the same animals was markedly higher, compared with that in the control rats (Figure 2(a)).

### 3.3. Element Levels in the Plasma

The exposure to SMV led to a distinct elevation of the Ca_p_ and K_p_ concentrations (Figure 1(b)), produced a visible decrease in the plasma Cu_p_ level (Figure 1(d)), and increased the plasma V_p_ concentration (Figure 1(e)). However, no SMV intoxication-related effects on the concentrations of Mg_p_, Na_p_, Cl_p_, and Zn_p_ (Figures 1(b), 1(c), and 1(d)) in the plasma were detected.

### 3.4. Excretion of Urinary Elements

In the SMV-intoxicated animals, Mg_u_, Ca_u_, and V_u_ were excreted in urine in higher amounts than those found in the control rats (Figures 2(c), 2(f), 3(c), and 3(f)). The urinary Cl_u_ excretion (per Cre_u_/24 h) also tended to be higher, compared with the control (Figure 3(e)). In turn, the urinary excretion of Zn_u_ (per 24 h and per Cre_u_/24 h) distinctly decreased in the SMV-exposed rats, in comparison with the control animals (Figures 2(d) and 3(d)), whereas the urinary K_u_ excretion (per 24 h, Figure 2(e), and per Cre_u_/24 h, Figure 3(e)) dropped and did not change, respectively. However, the urinary levels of Cu_u_ and Na_u_ (per 24 h and per Cre_u_/24 h), in the SMV-exposed rats, did not turn out to be significantly altered, compared with the control animals (Figures 2(d), 2(e), 3(d), and 3(e)).

### 3.5. Concentrations of Elements in the Kidney and FD

The exposure to SMV elevated the concentrations of Mg_k_, K_k_ (Figures 4(b) and 4(d)), and V_k_ (Figure 4(e)) and decreased the concentration of Cu_k_ (Figure 4(c)) in the kidney, compared with the control. A clear tendency toward decreased and increased concentrations of Ca_k_ and Zn_k_, respectively (Figures 4(b) and 4(c)), was also observed in the SMV-intoxicated animals, in comparison with the control rats. However, the concentration of Na_k_ in the same organ of the SMV-exposed rats did not change markedly, compared with the control (Figure 4(d)). In turn, the concentrations of Mg_FD_ and Ca_FD_ in FD decreased (Figures 5(a) and 5(b)), whereas the concentration of V_FD_ in the same FD increased (Figure 5(c)) after the SMV intoxication, in comparison with the control.

### 3.6. Kidney Weight and Renal MDA and TAS Levels

The SMV-exposed rats had a significantly elevated relative renal weight (RRW) and the renal MDA_k_ level, compared with the control animals (Figure 4(a)). The renal TAS_k_ level in the same group of rats was also distinctly enhanced, in comparison with the control (Figure 4(a)).

### 3.7. Correlations between Some Measured Variables

Significant positive and negative correlations and trends toward them were revealed between many different parameters, among others, between the renal V_k_ concentration, the renal MDA_k_, or TAS_k_ levels and some biomarkers and basic indices as well as between both OS markers (Table 3). They were also found between the fluid intake, BW, EUV, or the urinary Cre_u_ excretion and some investigated parameters (Table 3) as well as between the renal V_k_ concentration or the renal MDA_k_ level and the levels/concentrations of elements in the plasma, urine, kidney and FD (Table 4). The relationships between the urinary excretion of some elements and Cre_u_ or EUV were also indicated (Table 4).

## 4. Discussion

To our knowledge, the present report is the first to describe in a rat model the influence of the 12-week SMV exposure on the levels/activities of a wider panel of biomarkers of renal toxicity and on the concentrations of some elements in the kidney. It reveals a probable mechanism of the development of the SMV-induced functional renal disorders and proposes potential biomarkers of nephrotoxicity for specific conditions of SMV intoxication. It also illustrates for the first time many relationships between some explored indices. Because of different experimental conditions used, comparison of the results presented in the current report with literature data was difficult; therefore, the discussion section relies predominantly on our own results.

The increase in the kidney/body weight ratio observed by us in the present (Figure 4(a)) and previous study [28] in the SMV-intoxicated rats may indicate that V accumulated sufficiently in the kidney to manifest significant alterations in the size of this organ. Taking into account the fact that one of the main excretory routes for V within the body is the urinary system [29], V accumulation in the kidney may result in not only functional but also structural damage. Therefore, the estimation of the structural renal injury by histological examination in the rats after the 12-week SMV exposure is going to be performed in the nearest future. In this report, the degree of kidney damage has been evaluated only biochemically.

The decreased CreC together with the enhanced plasma Cre_p_ and U_p_ levels (Figure 1(a)), and the lowered urinary Cre_u_ and U_u_ excretion (Figure 2(a)) found in the SMV-exposed rats may indicate a problem with removal of Cre and U from the body and implicate glomerular functional disorders reflected by decreased clearance of the above-mentioned compounds. However, the lowered basic parameters such as fluid intake (discussed by us more widely) [30] and BW found in the same group of animals (Table 2) and the significant correlations between them and Cre_u_, Cre_p_, CreC, U_u_, and/or U_p_ (Table 3) do not allow us to exclude the possibility that dehydration and/or low BW affected the measured standard renal biomarkers. However, it is difficult to recognize to what extent they could influence the alterations in the biomarkers examined. The levels of Cre and U in the blood/urine are known to be affected not only by renal but also by some nonrenal factors [31–33]. On the other hand, the correlations between the renal V concentration (V_k_) and Cre_u_, Cre_p_, CreC, U_u_, and U_p_ (Table 3) confirm the involvement of V in the changes in the basic biomarkers explored. Moreover, the correlations between V_k_ and both OS markers such as MDA_k_ and TAS_k_ as well as between MDA_k_ and Cre_u_, Cre_p_, CreC, and U_u_ (Table 3) also point to the involvement of the SMV-induced OS in their alterations observed. Additionally, the positive correlation between both OS markers allows us to definitively conclude that the changes in the renal TAS_k_ level (Table 3) are associated with the alterations in LPO in the kidney due to the SMV exposure.

The markedly elevated urinary excretion of CysC_u_ (a tubular injury biomarker) [20, 32] demonstrated in the SMV-intoxicated rats (Figures 2(a) and 3(a)) without significant alterations in the plasma level of this protein (CysC_p_) (Figure 1(a)) may point to decreased reabsorption of CysC by injured tubules. Moreover, the positive correlation of CysC_u_ with TAS_k_ as well as the clear trends toward the positive correlations of CysC_u_ with V_k_ and MDA_k_ (Table 3) can suggest involvement of the renal V prooxidative action in the proximal tubular dysfunction (PTD), which consequently led to the urinary CysC wasting. It has been reported that when the renal proximal tubule remains intact CysC is completely taken up by the proximal tubular cells (PTC) [32], but in the case of tubular disease and the proximal convoluted tubule (PCT) injury the concentration of this protein in the urine is significantly elevated [34, 35]. In addition, the markedly enhanced urinary KIM-1_u_ excretion (Figure 3(a)) found in the same group of rats may also point to damage to PTC due to the SMV exposure. KIM-1 is expressed at very high levels in proximal tubule epithelial cells after toxic injury and it is present in urine when the injury of PTC occurs [20].

In turn, the lowered urinary activities of LDH_u_, ALP_u,_ GGTP_u_, and NAG_u_, demonstrated in the SMV-exposed rats (Figure 2(b)), allow us to suggest that the use of these tests for monitoring kidney injury during the SMV intoxication may not be valid, as the activity of these enzymes in the urine usually rises due to release from damaged cells. The lowered urinary ALP_u_ and GGT_u_ excretion and the simultaneous increase in the urinary LDH_u_ and NAG_u_ excretion were demonstrated by De la Torre et al. [36] in adult rats after vanadate intoxication. The discrepancies between our results concerning the urinary excretion of LDH and NAG and the results obtained by the above-mentioned investigators draw attention to the dependence of the renal effects on the conditions of vanadate exposure. In turn, the significant negative correlations found between the renal V_k_ concentration and the renal LDH_k_, ALP_k_, and GGTP_k_ activities (Table 3) may indicate a direct V impact. The direct inhibitory effect of both vanadate and vanadyl on the ALP activity was described by Cortizo et al. [37]. However, the marked increase in the plasma LDH activity (LDH_p_) demonstrated in the SMV-exposed rats (Figure 1(a)) requires further analyses. The measurement of the LDH isoenzyme might be the next important point in a more precise explanation of the significant increase in the activity of this enzyme in the plasma because the total plasma LDH is a highly sensitive test but, simultaneously, it is nonspecific. At the present stage of our study, we may only confirm the involvement of V and the SMV-induced OS in the changes in the activity of LDH in the blood, as evidenced by the significant positive correlations of LDH_p_ with V_k_ and MDA_k_ (Table 3).

Additionally, the strong positive correlations of the urinary pH with V_k_ and with both OS markers (Table 3) and the correlation between the renal V_k_ and K_k_ concentration and between the renal V_k_ concentration and the plasma K_p_ level (Table 4) also suggest involvement of OS in the mechanism of the rise in urine pH under the conditions of the SMV exposure (Figure 2(a)). They also confirm the dependence between the accumulation of V in the kidney and disorders in K homeostasis (Figures 1(b) and 4(d)). All these results suggest further measurements to elucidate the mechanism(s) of the changes observed in our experimental conditions.

The urinary Mg wasting (Figures 2(c) and 3(c)) and its elevated and diminished concentration in the kidney and FD, respectively (Figures 4(b) and 5(a)), as well as the clearly enhanced plasma and urinary Ca level (Figures 1(b) and 2(c)) and its lowered FD concentration (Figure 5(b)) point to disorders in the homeostasis of both macroelements during the SMV exposure. These changes may be partly explained by the influence of SMV on the bone tissue which is the main site of V uptake and deposition and in which the level of this element has been found to be significantly higher (Figure 5(c)). The presence of V in the bone might lead to mobilization of Mg and Ca from this tissue and cause the alterations observed. The negative correlations between the FD V, Mg, and Ca concentrations (Table 4) confirm the connection between the bone V accumulation and the bone Mg and Ca imbalance. Moreover, the negative correlation of V_k_ and MDA_k_ with the Mg_FD_ concentration and the clear trend toward this correlation between V_k_ and MDA_k_ with Ca_FD_ concentration (Table 4) also allow us to conclude that disturbances in bone Mg and Ca homeostasis observed in the rats at the SMV-exposure are associated both with the rise in the renal V accumulation and with the SMV-induced OS in the kidney. The changes in the levels of the other elements in biological fluids and in the kidney were also modified in correlation with the renal V_k_ concentration and/or with the SMV-intensified LPO in the kidney (Table 4).

Studies on the mechanisms of V action have shown that, as a transition metal, it may generate reactive oxygen species (ROS) and/or free radicals (FRs) and stimulate LPO or indirectly modify OS in cells, by releasing FR-generating metals from tissues, modifying enzymatic and antioxidant defence, or interacting with mitochondria [1, 38–40]. The literature data [1, 41] have revealed that OS may be involved in the mechanisms of the toxic action of vanadate. The correlations or clear trends toward them between the basic indices explored, some biomarkers of renal toxicity, or elements and the renal MDA_k_ and/or TAS_k_ levels (Table 3) suggest that in our experimental conditions the mechanism of the deleterious action of vanadate (as SMV) was associated with generation of OS in the kidney, as evidenced by the SMV-induced increase in both OS markers in this organ.

Finally, the lowered urinary excretion of Cre_u_, U_u_, UA_u_, KIM-1_u_, LDH_u_, GGTP_u_, ALP_u_, NAG_u_, and K_u_, when normalized per 24 h (Figure 2), and the unaltered or enhanced levels/activities of U_u_, UA_u_, TP_u_, ALB_u_, *β*_2_M_u_, LDH_u_, NAG_u_, and some elements in the urine, when normalized per Cre_u_/24 h (Figure 3), might be in part a consequence of the reduced EUV or the lowered urinary Cre_u_ excretion (Figure 2(a)), respectively, as indicated by the correlations of EUV or Cre_u_ with the above-mentioned indices (Tables 3 and 4).

## 5. Conclusions

The results of this preliminary study demonstrated that the exposure to SMV led to alterations in the levels/activities of some examined biomarkers of nephrotoxicity and caused renal mineral imbalance. They also pointed to a contribution of SMV-induced OS in the mechanism underlying the changes. Moreover, our findings revealed that the use of standard biomarkers of renal toxicity, such as Cre and U, and calculation of CreC for evaluation of kidney function at the SMV-induced fall in the fluid intake and BW may have a weak diagnostic value, since CreC and the plasma and urinary levels of Cre and U are greatly influenced by both the indices mentioned. In addition, the results simultaneously showed that the use of other classical enzymatic tests such as LDH_u_, ALP_u_, GGTP_u_, and NAG_u_ might not be valid either, due to their reduced urinary activities. Our results suggest that, from among the biomarkers tested, CysC_u_ and KIM-1_u_ might be the most appropriate in monitoring kidney function at the SMV exposure. The elevated urinary levels of both compounds may point to the proximal tubular dysfunction. Finally, for the first time our results also revealed many significant relationships between the parameters explored.

## Figures and Tables

**Table 1:** Operating parameters of the atomic absorption spectrometer with details of measurement of the levels of elements in biological samples as well as the certified and determined values of elements for the selected Certified Reference Materials (CRMs).

Parameters	Elements						
	V	Cu	Mg	Ca	Zn	Na	K
Technique	GF-AAS	GF-AAS	F-AAS				
FT	—		Air-acetylene				
FF (L/min)	Argon with the flow rate of 200 (mL/min) in all the steps except the atomization stage when the flow rate was 30 (mL/min)		1.8				
GTT	PCGTs (PyroTube CHR)		—				
SV (µL)	20 (*µL*)		—				
SM	B-CIA		B-CIM				
LC (mA)	10	7.5	7.5	10	5.0	10.0	10.0
WL (nm)	318.4	324.8	285.2	422.7	213.9	589.0	766.5
SW (nm)	1.3	1.3	1.3	0.2	1.3	0.2	1.3
DL (LOD)	K: 0.11 (µg/L)	K: 0.28 (µg/L)	K: 1 × 10^−5^ (mg/L)	K: 0.09 (mg/L)	K: 1 × 10^−3^ (mg/L)	K: 3.1 × 10^−3^ (mg/L)	K: 5.5 × 10^−3^ (mg/L)
	U: 0.23 (µg/L)	U: 0.14 (µg/L)	U: 6 × 10^−5^ (mg/L)	U: 0.36 (mg/L)	U: 0.6 (µ*g*/L)	U:**	U:**
	P: 0.23 (µg/L)	P: *√*	P: *√*	P: *√*	P: *√*	P:**	P:**
	FD: 0.47 (µg/L)		FD: 8 × 10^−4^ (mg/L)	FD: 0.13 (mg/L)			
LOQ	K: 0.33 (µg/L)	K: 0.84 (µg/L)	K: 3 × 10^−5^ (mg/L)	K: 0.27 (mg/L)	K: 3 × 10^−3^ (mg/L)	K: 9.3 × 10^−3^ (mg/L)	K: 16.5 × 10^−3^ (mg/L)
	U: 0.69 (µg/L)	U: 0.42 (µg/L)	U: 1.8 × 10^−4^ (mg/L)	U: 1.08 (mg/L)	U: 1.8 (µ*g*/L)	U:**	U:**
	P: 0.69 (µg/L)	P: *√*	P: *√*	P: *√*	P: *√*	P:**	P:**
	FD: 1.41 (µg/L)		FD: 2.4 × 10^−3^ (mg/L)	FD: 0.39 (mg/L)			
CV (%)	0.2–2	0.5–2	0.5–1				
							

Elements	Bovine Liver 1577 c			Seronorm Trace Elements Urine 201205		Trace Elements in Natural Water 1640 a	
	Certified value	Determined value^§^		Certified value	Determined value^§^	Certified value	Determined value^§^

Mg	620 ± 42 (mg/kg)	657.4 ± 20.2 (mg/kg)		71.1 ± 2.5 (mg/L)	69.7 ± 2.3 (mg/L)	1.058 ± 0.0040 (mg/L)	1.059 ± 0.040 (mg/L)
Ca	131 ± 10 (mg/kg)	133.4 ± 15.2 (mg/kg)		111 ± 2 (mg/L)	111.9 ± 1.2 (mg/L)	5.615 ± 0.021 (mg/L)	5.649 ± 0.028 (mg/L)
V	8.17 ± 0.66 (*µg*/kg)	10.2 ± 1.6 (*µg*/kg)		25.2 ± 1.4 (µg/L)	22.43 ± 0.56 (µg/L)	12.99 ± 0.37 (µg/L)	12.33 ± 0.35 (µg/L)
Zn	181.1 ± 1.0 (mg/kg)	180.3 ± 8.7 (mg/kg)		1141 ± 79 (µg/L)	1100.6 ± 24.4 (µg/L)	55.64 ± 0.35 (µg/L)	55.50 ± 0.17 (µg/L)
Cu	275.2 ± 4.6 (mg/kg)	268.6 ± 4.7 (mg/kg)		78 ± 8 (µg/L)	92.4 ± 6.9 (µg/L)	85.75 ± 0.51 (µg/L)	85.45 ± 0.14 (µg/L)
Na	2.033 ± 0.064 (mg/kg)	1.969 ± 0.015 (mg/kg)		2307 ± 56 (mg/L)	2376 ± 45 (mg/L)	3.137 ± 0.031 (mg/L)	3.150 ± 0.025 (mg/L)
K	—	—		1903 ± 42 (mg/L)	1930 ± 40 (mg/L)	0.5799 ± 0.0023 (mg/L)	0.5818 ± 0.0209 (mg/L)

Elements	Bone Ash 1400						
		Certified value				Determined value^§^	

Mg		6.84 ± 0.13 (mg/g)				6.774 ± 0.065 (mg/g)	
Ca		381.8 ± 1.3 (mg/g)				384.8 ± 2.2 (mg/g)	

FT: flame type; FF: fuel flow; GTT: graphite tube type; SV: sample volume; SM: signal mode; LC: lamp current; WL: wavelength; SW: slit width; PCGTs: pyrolytically coated graphite tubes; B-CIA: background-corrected integrated absorbance; B-CIM: background-corrected integral mode.

^*√*,∗∗^Determined colourimetrically or by an EasyLyte analyser, respectively.

DL (LOD): detection limit.

LOQ: quantification limit.

CV: coefficient of variation.

K: kidney; U: urine; P: plasma; FD: femoral diaphysis.

^§^Mean ± SD, *n* = 5.

**Table 2:** Basic indices in the tested animal groups at week 12.

Parameters	Groups of animals		Percentage decrease (↓) compared with Group I, *P* value
	(I) Control	(II) SMV	
Fluid intake (mL/rat/24 h)^2^	58.53 ± 1.92	39.64 ± 0.73*	↓ 32 < 0.001
Food intake (g/rat/24 h)^1^	37.02 ± 0.86	28.73 ± 0.69*	↓ 22 < 0.001
Body weight (% of initial b. wt.)^1^	264.13 ± 23.59	190.46 ± 15.95*	↓ 28 < 0.05
V dose (mg V/kg b·wt./24 h)^#^	—	13.27 ± 0.33	

^1,2^Data were tested by Student's *t*-test or Welch's *t*-test, respectively.

*Significant differences (*P* < 0.05), compared with the Control (Group I).

^#^Consumed with drinking water.

**Table 3:** Correlation coefficients for compared variables.

Variables							
V_k _			MDA_k _			TAS_k_	
Food-I	−**0.894^†^**		Food-I	−**0.817^†^**		Food-I	−**0.676^†^**
Fluid-I	−**0.905^†^**		Fluid-I	−**0.754^†^**		Fluid-I	−**0.707^†^**
RRW	**0.778^†^**		RRW	0.531*		RRW	***0.406*** ^ f^
BW	−0.601*		BW	−**0.672^†^**		BW	−**0.780^†^**
MDA_k_	**0.889^†^**		TAS_k_	0.598*		LDH_k_	−***0.472*** ^a^
TAS_k_	0.590*		LDH_k_	−0.614*		ALP_k_	−***0.492*^†††^**
LDH_k_	−0.544*		ALP_k_	−**0.757^†^**		GGTP_k_	−***0.401*** ^g^
ALP_k_	−**0.669^†^**		GGTP_k_	−0.577*		EUV	−***0.412*** ^f^
GGTP_k_	−**0.759^†^**		U_k_	−***0.445*** ^c^		pH	0.588*
EUV	−**0.716^†^**		ALB_k_	−0.549*		Cre_p_	**0.624^†^**
pH	**0.917^†^**		EUV	−0.613*		CreC	−0.505*
Cre_u_	−**0.630^†^**		pH	**0.761^†^**		U_u_′	−**0.*429*** ^d^
U_p_	0.483**		Cre_p_	**0.757^†^**		CysC_u_′	0.601*
Cre_p_	**0.818^†^**		CreC	−0.606*		β_2_M_u_′	−***0.408*** ^h^
CreC	−**0.652^†^**		TP_p_	−***0.437*** ^d^		AlP_u_′	−0.552*
ALP_p_	−***0.448*** ^c^		LDH_p_	0.539*		NAG_u_′	−***0.448*** ^c^
LDH_p_	**0.757^†^**		Cre_u_	−0.572*			
U_u_′	−0.596*		U_u_′	−0.574*			
TP_u_′	−**0.654^†^**		TP_u_′	−0.518*			
CysC_u_′	***0.399*** ^ g^		CysC_u_′	***0.496*** ^ f^			
LDH_u_′	−0.612*		LDH_u_′	−0.523*			
*AL*⁡P_u_′	−**0.656^†^**		*AL*⁡P_u_′	−0.514*			
GGTP_u_′	−***0.531*** ^a^		GGTP_u_′	−***0.425*** ^i^			
NAG_u_′	−0.506*		NAG_u_′	−***0.490*** ^ff^			

Fluid-I		BW		EUV		Cre_u_	

EUV	**0.759^†^**	Cre_p_	−**0.773^†^**	Cre_u_	**0.920^†^**	U_u_′′	−0.561*
Cre_p_	−**0.844^†^**	Cre_u_	0.595*	U_u_′	**0.816^†^**	UA_u_′′	−***0.443*** ^c^
Cre_u_	**0.668^†^**	CreC	**0.762^†^**	UA_u_′	0.499*	TP_u_′′	−**0.786^†^**
CreC	**0.755^†^**	U_u_′	**0.734^†^**	LDH_u_′	0.569*	*AL*⁡B_u_′′	−**0.630^†^**
U_p_	−***0.470*** ^a^	CysC_p_	***0.488^‡‡^***	*AL*⁡P_u_′	***0.424*** ^ e^	β_2_M_u_′′	−**0.697^†^**
U_u_′	**0.700^†^**	CysC_u_′	−***0.482*** ^a^	GGTP_u_′	0.643*	LDH_u_′′	−**0.776^†^**
				NAG_u_′	**0.780^†^**		
				KIM-1_u_′	***0.447*** ^ c^		

Data are presented as the correlation coefficients (*r*) and the levels of statistical significance (*P*).

^p,u,k^Plasma, urine, and kidney (concentration), respectively.

Fluid-I, Food-I, RRW, BW, and EUV: fluid intake, food intake, renal relative weight, body weight, and excreted urinary volume, respectively.

^†^ *P *< 0.01; *P < 0.05; ^f^ *P *= 0.051; ^†††^ *P *= 0.053; ^ff^ *P *= 0.054; ^‡‡^ *P *= 0.055; **P = 0.058; ^a^ *P *= 0.06; ^c^ *P *= 0.08; ^d^ *P *= 0.09; ^e^ *P = *0.10; ^f^ *P = *0.11; ^g^ *P = *0.12; ^h^ *P = *0.13; ^i^ *P = *0.14.

^′,′′^Expressed per 24 h and per Cre_u_ per 24 h, respectively.

The significant correlations and tendencies toward them are highlighted in normal and italic bold font, respectively.

**Table 4:** Correlation coefficients for measured variables.

	Variables	
	V_k_	MDA_k_
V_p_	** 0.872^†^**	** 0.710^†^**
Cu_p_	−0.520*	−***0.445*** ^b^
K_p_	0.621*	—
V_u_′	** 0.977^†^**	**0.795^†^**
Mg_u_′	**0.798^†^**	**0.678^†^**
Zn_u_′	−0.555*	−0.618*
K_u_′	−0.600*	−0.535*
Cu_k_	−**0.687^†^**	−**0.628^†^**
Mg_k_	0.527*	—
K_k_	0.599*	—
V_k_	—	** 0.889^†^**
Ca_k_	−***0.486*** ^#^	−***0.479*** ^a^
Mg_FD_	−**0.767^†^**	−**0.725^†^**
Ca_FD_	−***0.492*^*∂*^**	−***0.334*** ^c^

	V_FD_	

Mg_FD_	−0.768*	
Ca_FD_	−0.547*	

	EUV	

V_u_′	−**0.722^†^**	
K_u_′	** 0.856^†^**	

	Cre_u_	

V_u_′′	−0.600*	
Mg_u_′′	−0.602*	
Ca_u_′′	−**0.633^†^**	
Cu_u_′′	−**0.688^†^**	
K_u_′′	−0.540*	
Cl_u_′′	−**0.735^†^**	

Data are presented as the correlation coefficients (*r*) and the levels of statistical significance (*P*).

^p,u,FD,k^Plasma, urine, femoral diaphysis, and kidney (concentration), respectively.

EUV: excreted urinary volume.

^†^ *P *< 0.01; *P < 0.05; ^*∂*^ *P =* 0.053; ^#^ *P =* 0.056; ^a^ *P *= 0.061; ^b^ *P *= 0.08; ^c^ *P *= 0.20.

^′, ′′^Expressed per 24 h and per Cre_u_ per 24 h.

The significant correlations and tendencies toward them are highlighted in normal and italic bold font, respectively.

## Endnotes

1. They were bought as part of the Project entitled “Building of the Center for Interdisciplinary Research” realized within the frame of the Operating Programme “Development of Eastern Poland” 2007–2013, Priority I: Modern Economy, Action I.3. The Advancement of Innovation, cofinanced by the European Regional Development Fund.
